# Baseline mucosal transcriptomic endotypes stratify healing capacity and biologic therapy outcomes in ulcerative colitis: an integrative multi-cohort study

**DOI:** 10.3389/fphar.2026.1931118

**Published:** 2026-10-23

**Authors:** Wenbiao Chen, Shiyong Zeng

**Affiliations:** 1 School of Medicine, Quanzhou Medical College, Quanzhou, Fujian, China; 2 Key Laboratory of Screening and Control of Infectious Diseases (Quanzhou Medical College), Quanzhou, Fujian, China

**Keywords:** biologic therapy, endotypes, integrative transcriptomics, mucosal healing, transcriptomics, ulcerative colitis

## Abstract

**Background:**

Numerous baseline mucosal transcriptomic signatures have been proposed to stratify therapeutic outcomes in ulcerative colitis (UC), but cross-cohort transferability is frequently undermined by platform heterogeneity, endpoint/timepoint differences, and drug-specific biases. The resulting lack of a biologic-agnostic molecular baseline has limited clinical translation and prospective trial design.

**Methods:**

We integratively re-analyzed endpoint-linked baseline mucosal transcriptomes from seven public UC GEO series (778 unique baseline samples), with three additional public accessions used for disease-activity and contextual analyses. The primary response/healing synthesis used five study slices spanning anti-TNF, anti-integrin, and etrolizumab-treated cohorts. Unsupervised decomposition identified baseline endotypes, and endotype-outcome associations were estimated within each cohort (OR per 1-SD increase) and summarized by random-effects cross-cohort pooling with heterogeneity reporting, rank/seed sensitivity analyses, endpoint-restricted summaries, and negative controls.

**Results:**

Across five primary response/healing study slices, an epithelial barrier endotype (EPITHELIAL_BARRIER/NMF_C3) was associated with higher odds of response/healing (random-effects cross-cohort pooling OR = 3.171, 95% CI 1.491–6.744), whereas macrophage-inflammation and lymphoid/complement-stromal axes (MACROPHAGE_INFLAMMATION/NMF_C1 and LYMPHOID_COMPLEMENT_STROMAL/NMF_C2) were associated with lower odds of response/healing (OR = 0.359, 0.175–0.735; OR = 0.378, 0.220–0.651). The epithelial estimate was largest in GSE73661 but remained significant in the five-study leave-one-out summary after excluding that cohort (OR = 2.547, 95% CI 1.271–5.105). Cross-mechanism evaluation included UNIFI ustekinumab week-8 mucosal healing (UST arm n = 358; EPITHELIAL_BARRIER OR = 4.483, 2.263–8.881) and directionally consistent effects in etrolizumab. Endotype axes were supported by independent single-cell localization (FDR = 0.0026). Treatment-interaction tests were imprecise and did not establish drug-class-specific prediction. In cohort-heldout prediction, AUC ranged from 0.717 to 0.758, but calibration was variable. A network-based therapeutic prioritization was enriched for UC-approved agents (AUC = 0.741; permutation p = 6.0 × 10^−^^4^) and is presented as hypothesis-generating.

**Conclusion:**

Baseline mucosal endotypes stratify healing capacity and therapeutic outcomes across public cohorts, with orthogonal support from single-cell localization. These findings support prospective evaluation as biologic-agnostic outcome stratifiers rather than established drug-specific predictive biomarkers.

## Introduction

Despite the expanding therapeutic armamentarium for ulcerative colitis (UC), achieving steroid-free mucosal healing remains a challenge, and biologic selection is often empirical. Treat-to-target strategies emphasize early assessment and endoscopic goals, yet baseline stratifiers that generalize between cohorts and therapies remain limited (Turner et al., 2021; Peyrin-Biroulet et al., 2015). Transferable baseline stratifiers could enable biologic-agnostic risk enrichment, support rational sequencing strategies, and improve the design of prospective biomarker-guided trials, but require cross-cohort evaluation with conservative interpretation.

The barrier and mucus layer of the colonic epithelium, together with inflammatory niches enriched for myeloid and stromal programs, repeatedly emerge as key axes of UC mucosal remodeling (van der Post et al., 2019; Kondo et al., 2021; Du et al., 2023). Inflammatory myeloid and stromal programs also recur in treatment-response studies, including OSM-linked refractory biology (Garrido-Trigo et al., 2023; Arijs et al., 2018; Gazouli et al., 2022). However, baseline signatures often degrade when carried to cohorts measured on different platforms, with different endpoints, or across mechanisms of action (Verstockt et al., 2019; Telesco et al., 2018; Pavlidis et al., 2022). Myeloid and stromal states, in particular, have been linked to refractory behavior and response variation across studies, reinforcing the need to interpret bulk signatures in a compartment-aware manner (Verstockt et al., 2021; Dharmasiri et al., 2021).

One practical obstacle is that bulk biopsies are mixtures whose cellular composition and activation state shift with inflammation and treatment history. As a result, cohort-specific differences in sampling site, endpoint window, and platform can amplify compositional effects and obscure transferable mucosal programs. High-resolution single-cell and spatial studies help define the cellular provenance of these programs and provide a benchmark for interpreting bulk signatures (Parikh et al., 2019; Mennillo et al., 2024; Thomas et al., 2024). Related single-cell atlases further show how epithelial and immune-cell states vary across inflamed intestinal tissue (Smillie et al., 2019; Huang et al., 2019; Corridoni et al., 2020). Complementary tissue profiling and model systems, including multiplexed imaging and patient-derived organoids, can refine compartmental interpretations, but causal attribution still requires perturbation or prospective validation (Haberman, 2020; Chen et al., 2021; Geng et al., 2018). Endoscopy-linked molecular profiling and imaging-supported biomarker approaches also provide a clinically aligned lens for connecting mucosal programs to decision-relevant endpoints (Iacucci et al., 2023; Iacucci et al., 2021).

We therefore focused on extracting coherent baseline programs that recur across studies and mapping them to clinically relevant outcomes using conservative cross-cohort evidence. Real-world uptake of treat-to-target recommendations remains limited, further increasing the need for baseline stratification tools that generalize across settings (Bryant et al., 2018).

We integrated public baseline mucosal transcriptomic cohorts across biologic mechanisms to identify baseline endotypes and quantify associations with response/healing using within-cohort effect sizes and random-effects cross-cohort pooling. Endotype axes were evaluated for alignment with epithelial barrier/goblet and inflammatory myeloid programs using bulk program scoring and independent single-cell corroboration. Treatment-interaction, prediction, and therapeutic-prioritization analyses were used to contextualize clinical translation while avoiding claims of established drug-specific prediction (Figure 1).

**FIGURE 1:**
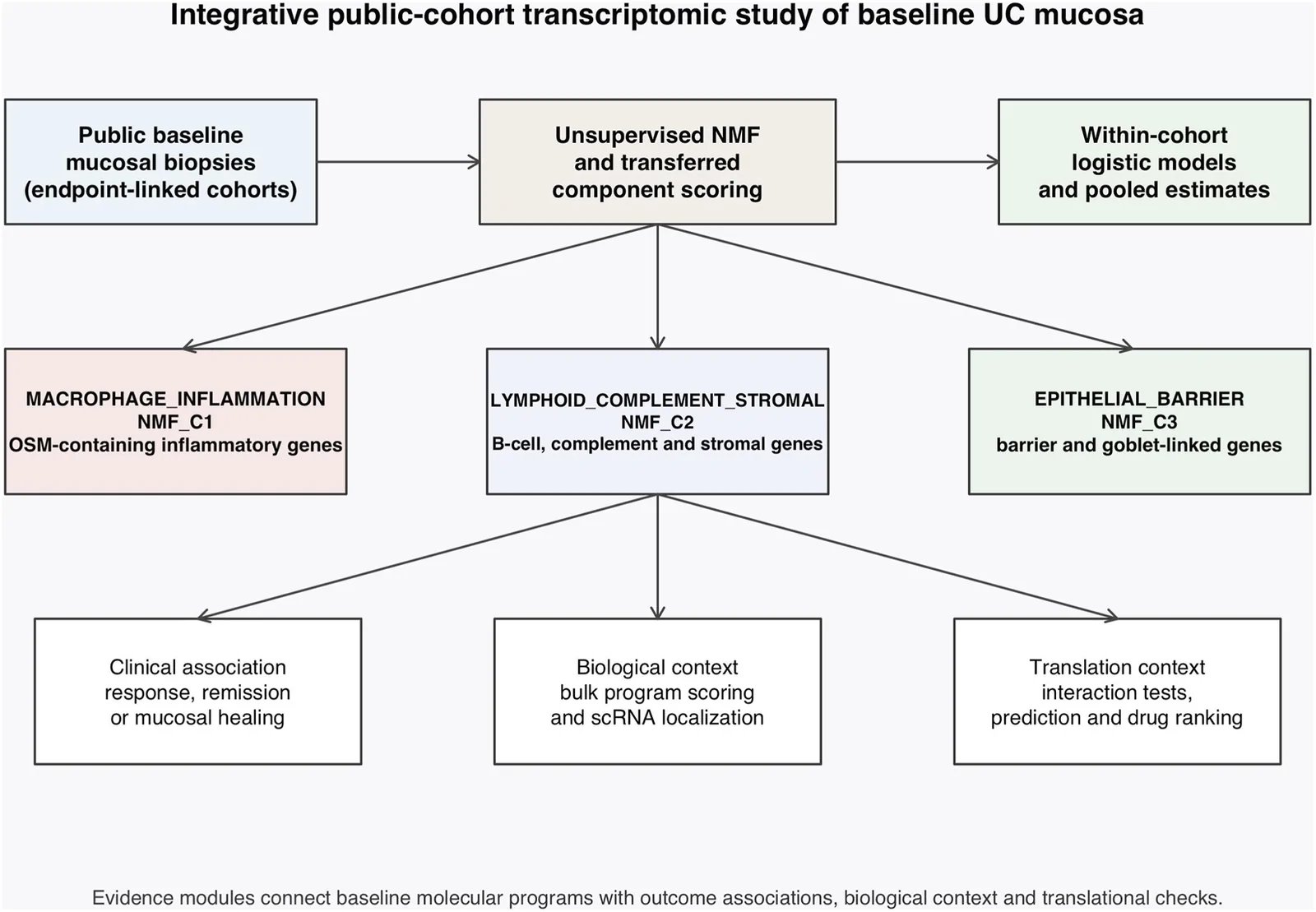
Mechanistic overview and study workflow. Baseline UC mucosal biopsies (week 0) are used to derive transferable baseline endotypes. Cohorts/endpoints are summarized in Table 1. Endotype–outcome associations are estimated within each cohort (OR per 1-SD increase in within-cohort standardized score) and summarized by random-effects cross-cohort pooling; biological localization is supported by bulk program scoring and independent single-cell corroboration. Therapeutic prioritization integrates network proximity and perturbation-signature reversal to nominate repurposing candidates for further evaluation.

## Materials and methods

### Study design

We analyzed de-identified public transcriptomic datasets of UC mucosal biopsies. Institutional review board (IRB) exemption was confirmed as no new human subjects research, patient recruitment, or intervention was performed.

### Datasets and endpoints

Public GEO datasets with baseline mucosal biopsies and subsequent response/remission or healing endpoints were selected (Table 1). When trials included active and placebo arms, analyses were stratified by arm when appropriate, including placebo-treated controls. Endpoints were defined as reported in the original studies and harmonized into binary outcomes within each cohort/timepoint for association analyses. Datasets were selected to reflect clinically decision-relevant endpoints, include multiple biologic mechanisms of action, and support cross-cohort synthesis, cross-mechanism evaluation, placebo-arm context, and endpoint-diversity checks.

**TABLE 1:** Study cohorts, therapies, endpoints, and baseline sample sizes.

Cohort (GEO)	Therapy/setting	Endpoint (timepoint)	Baseline n used	Event/non-event	Key covariates/notes
GSE73661	Mixed IFX/VDZ	Mucosal healing (W6)	47	11/36	Adjusted for Mayo endoscopic subscore; baseline W0 linked to W6; treatment-arm adjustment is reported as a sensitivity model where available. (Arijs et al., 2018)
GSE92415	Golimumab	Response (W6)	59	32/27	Baseline W0; includes placebo-treated controls in the original trial design and resampling-based negative controls in this analysis. (Sandborn et al., 2014)
GSE16879	Infliximab	Endoscopic/histologic response	24	8/16	UC colon, pre-IFX baseline; wide CI due to small n. (Arijs et al., 2009a)
GSE12251	Infliximab (ACT1)	Endoscopic + histologic healing (W8)	23	12/11	Independent IFX cohort for primary cross-cohort synthesis. (Arijs et al., 2009b)
GSE206285	UST (UNIFI)	Mucosal healing (W8)	358	56/302	UST arm baseline sigmoid biopsies; placebo analyzed separately. (Pavlidis et al., 2022)
GSE206285	UST (UNIFI)	Clinical remission (W8)	364	49/315	Orthogonal endpoint within the same dataset. (Pavlidis et al., 2022)
GSE72819	ETRO (EUCALYPTUS)	Clinical remission (W10)	27	9/18	Anti-TNF-naïve subset used for cross-mechanism context. (Tew et al., 2016)
GSE109142	Standardized pediatric therapy	Remission (W4)	206	105/101	Endpoint diversity check; pediatric baseline RNA-seq. (Haberman et al., 2019)

Selected cohorts included vedolizumab/mixed IFX-VDZ samples (GSE73661), golimumab (GSE92415), and infliximab cohorts (GSE16879 and ACT1/GSE12251) (Arijs et al., 2018; Sandborn et al., 2014; Arijs et al., 2009a; Arijs et al., 2009b). Cross-mechanism and endpoint-diversity analyses used ustekinumab (UNIFI/GSE206285), etrolizumab (EUCALYPTUS/GSE72819), and a pediatric cohort (GSE109142) (Pavlidis et al., 2022; Tew et al., 2016; Haberman et al., 2019).

Only baseline samples linked to a subsequent endpoint were included in the primary association analyses. Post-treatment samples, control samples, or repeated rows from other timepoints were excluded from baseline predictor models and retained only for contextual or negative-control analyses where explicitly indicated. When a public series did not provide a participant identifier, each baseline sample was treated as one independent endpoint-linked biopsy and this limitation was recorded in the harmonization file. Dose-arm information was included where available, most notably in UNIFI (UST 130 mg and 6 mg/kg arms), but within-patient dose escalation, therapeutic drug monitoring, anti-drug antibodies, and post-baseline dose changes were not consistently available in public metadata.

Baseline clinical covariates were inconsistently reported across public cohorts. Where severity proxies were available, we report baseline characteristics and quantify endotype–severity correlations as supplementary context (see Supplementary Tables S1 and S5).

### Endotype discovery and scoring

Non-negative matrix factorization (NMF) was applied to baseline bulk transcriptomes to identify latent molecular patterns (endotypes) representing coherent biological processes, yielding three components (Brunet et al., 2004). The primary NMF run used k = 3, seed = 1, multiplicative-update optimization, a maximum of 400 iterations, and convergence tolerance of 1 × 10^−4^. The rank k = 3 was selected because it produced interpretable epithelial, inflammatory myeloid, and lymphoid/complement-stromal axes and was supported by sensitivity checks across k = 2–4 and three random seeds (Supplementary Table S7; Supplementary_Tables_All.xlsx, Sheet Additional_file_10). Components were annotated by dominant program alignment and are referenced by their primary biological interpretation throughout: MACROPHAGE_INFLAMMATION (NMF_C1), LYMPHOID_COMPLEMENT_STROMAL (NMF_C2), and EPITHELIAL_BARRIER (NMF_C3). Component activity scores were computed per sample and standardized within cohort for association analyses. For cross-dataset transfer, each component was represented by its top 50 component genes; expression values were z-scored gene-wise within each dataset, and the sample-level component program score was calculated as the mean z-score across genes present in that component gene set.

### Association analyses and cross-cohort synthesis

Within each cohort, associations between standardized endotype scores and binary response/healing endpoints were estimated using logistic regression and reported as odds ratios (OR) with 95% confidence intervals (CI). Prespecified clinical covariates (e.g., Mayo endoscopic subscore, Mayo score, therapy/dose arm, age, or pediatric treatment category) were included where available. Because several cohorts were small, high-leverage estimates were additionally checked using Firth-type mean bias-reduced logistic regression; this was prespecified for sparse-event cohorts, with GSE73661 reported explicitly because only 11 of 47 baseline-linked samples achieved mucosal healing. Multiple testing was controlled using the Benjamini–Hochberg false discovery rate (FDR). Random-effects cross-cohort synthesis (DerSimonian–Laird) was used to summarize effects across primary studies, with heterogeneity quantified by I^2^ and τ^2^. Restricted maximum likelihood (REML) and endpoint-restricted summaries were added as sensitivity analyses.

### Robustness analyses and treatment interaction tests

Robustness was evaluated across NMF hyperparameters and random seeds, and the primary five-study evidence base was also summarized with leave-one-study-out random-effects estimates. Negative controls included label permutation and randomized subset resampling to assess the likelihood of spurious associations. In randomized trial datasets with both active and placebo arms, treatment x endotype interaction terms were used to probe treatment specificity versus baseline healing tendency. These interaction models compared active therapy with placebo and used within-cohort standardized endotype scores; UNIFI dose arms were also evaluated separately against placebo.

### Mechanistic localization and scRNA corroboration

Epithelial barrier/goblet and inflammatory myeloid programs were quantified in bulk tissue using program scoring. Independent corroboration was performed in a UC scRNA dataset (GSE231993) by comparing program scores between epithelial and myeloid compartments within paired samples using paired sign-flip exact testing, with FDR correction. Epithelial and myeloid compartments were defined by marker-count gating: cells were assigned to epithelial or myeloid compartments when the relevant marker count was at least two and at least 1.5-fold higher than the competing marker set; cells not meeting a unique dominance criterion were not used for the paired localization contrast. This scRNA module was designed to corroborate epithelial barrier/goblet and inflammatory myeloid localization. The LYMPHOID_COMPLEMENT_STROMAL component was interpreted from its bulk top genes and program overlap, including B-cell/lymphoid, complement, and stromal markers, rather than from a dedicated stromal-cell scRNA contrast. Program scoring provides a gene-set activity proxy similar to GSVA/GSEA (Hanzelmann et al., 2013; Subramanian et al., 2005).

### Therapeutic prioritization

Therapeutic hypotheses were prioritized using (i) network proximity between an endotype-associated disease module and drug targets (BioGRID interactions and DrugCentral targets) and (ii) perturbation signature reversal using CREEDS signatures. Matched negative controls and enrichment toward UC-approved therapies were used to assess non-randomness (Oughtred et al., 2021; Ursu et al., 2019; Qi et al., 2020).

### Prediction evaluation beyond ROC

Prediction performance within anti-TNF cohorts was evaluated using leave-one-cohort-out training/testing to reduce optimistic bias, reporting AUC with uncertainty, calibration summaries, and decision-curve analysis (Vickers and Elkin, 2006).

## Results

### Cohort landscape and endpoints

Publicly available UC mucosal transcriptomic cohorts with baseline biopsies and subsequent response/healing endpoints were analyzed across biologic mechanisms. Trial-like cohorts with baseline samples and binary endpoints comprised the primary evidence base (Table 1), with additional datasets or arms used for cross-mechanism evaluation, placebo-arm context, and endpoint-diversity checks.

Endpoint-linked baseline analyses included 778 unique baseline samples across seven public GEO series; three additional accessions contributed disease-activity or contextual transcriptomic comparisons. Event counts for the main endpoint-linked analysis slices ranged from 8/24 responders in the small infliximab cohort (GSE16879) to 56/358 week-8 mucosal healing events in the UNIFI UST arm (Table 1; Supplementary Table S6).

### Conserved baseline mucosal programs stratify response and mucosal healing across cohorts

Three baseline components were identified and annotated by dominant program alignment: MACROPHAGE_INFLAMMATION (NMF_C1), LYMPHOID_COMPLEMENT_STROMAL (NMF_C2), and EPITHELIAL_BARRIER (NMF_C3). Random-effects cross-cohort synthesis across five primary response/healing study slices supported EPITHELIAL_BARRIER as associated with improved outcomes (OR>1), whereas MACROPHAGE_INFLAMMATION and LYMPHOID_COMPLEMENT_STROMAL were associated with lower odds of response/healing (OR<1) (Table 2). Heterogeneity was low to moderate (I2 = 0–25.3%), consistent with broadly similar directions across cohorts despite endpoint and platform differences. REML sensitivity estimates were concordant with the primary DerSimonian-Laird summaries (EPITHELIAL_BARRIER OR = 2.964, 95% CI 1.515–5.799; MACROPHAGE_INFLAMMATION OR = 0.396, 0.233–0.673; LYMPHOID_COMPLEMENT_STROMAL OR = 0.378, 0.220–0.651; Supplementary Table S7). In an endpoint-restricted sensitivity analysis limited to endoscopic/histologic response or mucosal-healing endpoints (GSE73661, GSE16879, GSE12251, and UNIFI/GSE206285), associations remained directionally consistent: EPITHELIAL_BARRIER OR = 4.914 (95% CI 2.709–8.916), MACROPHAGE_INFLAMMATION OR = 0.266 (0.126–0.560), and LYMPHOID_COMPLEMENT_STROMAL OR = 0.414 (0.235–0.731). The largest single-cohort epithelial estimate occurred in GSE73661, where only 11 of 47 patients achieved mucosal healing; Firth-type bias-reduced logistic regression attenuated this estimate but preserved directionality (OR = 14.0, 95% CI 1.48–131.24). In the five-study leave-one-study-out summary, EPITHELIAL_BARRIER remained associated with higher odds of response/healing after excluding GSE73661 (OR = 2.547, 95% CI 1.271–5.105), supporting the pooled signal while emphasizing that individual-cohort odds ratios should be interpreted cautiously. For MACROPHAGE_INFLAMMATION, the pooled direction was retained after excluding GSE73661, but the estimate became less precise and crossed the null (OR = 0.299, 95% CI 0.087–1.024; p = 0.055; Supplementary_Tables_All.xlsx, sheet Additional_file_25). In downstream program scoring, EPITHELIAL_BARRIER aligned with barrier/goblet programs, while MACROPHAGE_INFLAMMATION and LYMPHOID_COMPLEMENT_STROMAL aligned with inflammatory and lymphoid/stromal mucosal remodeling programs (Figure 2).

**TABLE 2:** Random-effects cross-cohort synthesis of baseline endotypes (primary response/healing studies).

Endotype	Studies (k)	OR (95% CI)	p value	I^2^ (%)	τ^2^
MACROPHAGE_INFLAMMATION (NMF_C1)	5	0.359 (0.175–0.735)	0.0051	25.3	0.168
LYMPHOID_COMPLEMENT_STROMAL (NMF_C2)	5	0.378 (0.220–0.651)	4.45 × 10^−4^	0.0	0
EPITHELIAL_BARRIER (NMF_C3)	5	3.171 (1.491–6.744)	2.72 × 10^−3^	12.8	0.102

**FIGURE 2:**
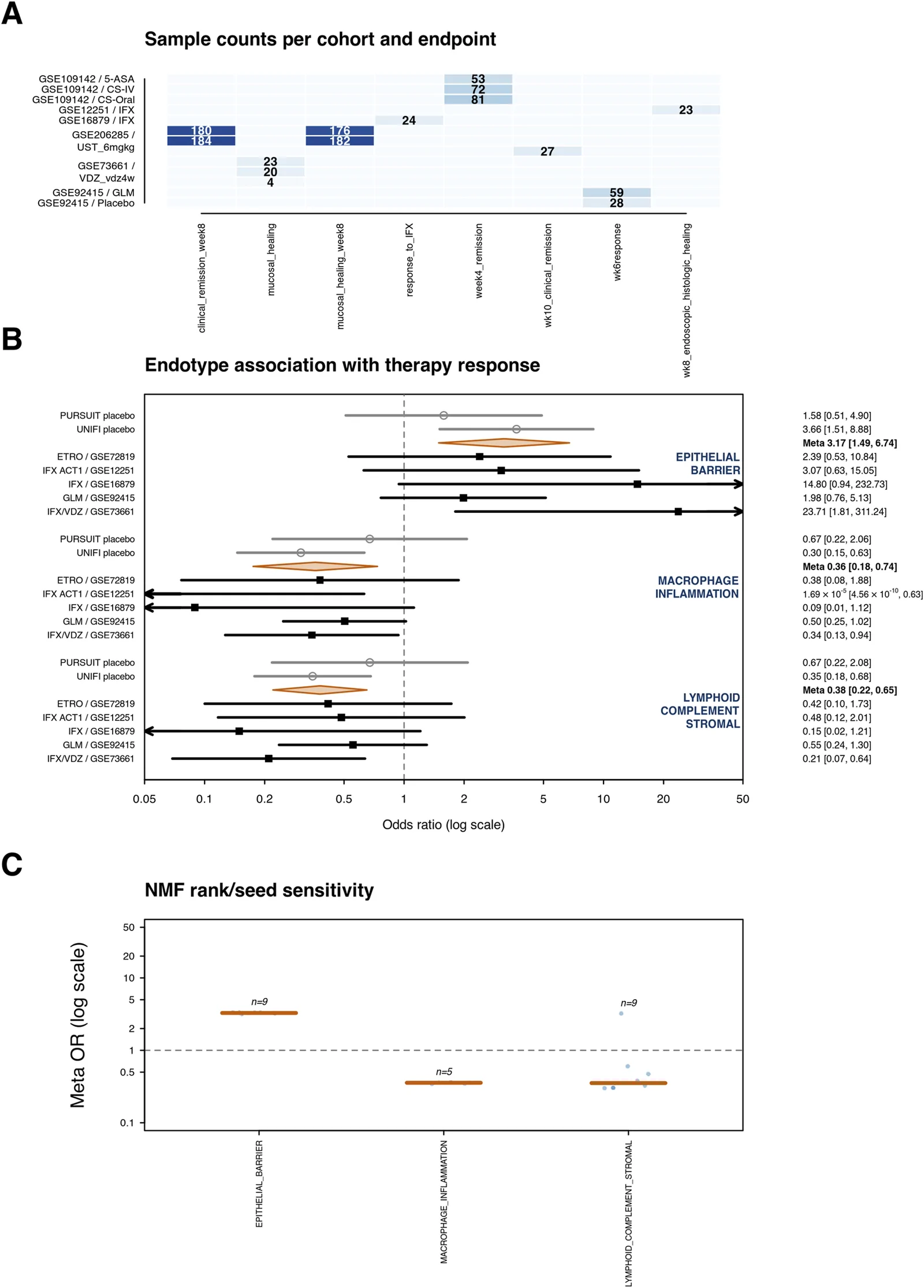
Transferable baseline endotypes and response/healing associations across cohorts. **(A)** summarizes cohorts and endpoints used for primary, placebo-control, cross-mechanism, and endpoint-diversity analyses (Table 1). **(B)** quantifies cohort-level endotype-outcome associations (logistic regression; OR per 1-SD increase in within-cohort standardized endotype score) and their random-effects pooled estimates (DerSimonian-Laird) across the primary evidence base (GSE73661, GSE92415, GSE16879, GSE12251, and GSE72819). Event/non-event counts for the plotted analysis slices are provided in Table 1, Supplementary Table S6, and Supplementary_Tables_All.xlsx, sheet Additional_file_23. Placebo-arm rows are shown separately as negative-control/prognostic context. **(C)** summarizes NMF rank/seed sensitivity across the same primary study-slice definition; the canonical five-study leave-one-study-out summary is provided in Supplementary_Tables_All.xlsx, sheet Additional_file_25.

### Generalization across mechanisms of action

Cross-mechanism evaluation included cohorts treated with distinct mechanisms of action (MOA), including an endpoint-rich UST cohort (UNIFI; baseline→week-8 mucosal healing and clinical remission) and an ETRO anti-beta7 cohort (EUCALYPTUS; baseline→week-10 clinical remission) (Pavlidis et al., 2022; Tew et al., 2016). The five-study primary synthesis included EUCALYPTUS, whereas UNIFI was used as a larger, endpoint-rich cross-mechanism context and in the endpoint-restricted healing sensitivity analysis. In UNIFI, EPITHELIAL_BARRIER (NMF_C3) showed strong associations with week-8 mucosal healing in the UST arm (OR = 4.483, 95% CI 2.263–8.881; FDR = 2.55 × 10^−5^; n = 358) and week-8 clinical remission (OR = 4.499, 95% CI 2.183–9.270; FDR = 6.84 × 10^−5^; n = 364). Conversely, MACROPHAGE_INFLAMMATION (NMF_C1) was associated with lower odds of healing (OR = 0.288, 0.170–0.489). Effects in the ETRO cohort showed a similar trend (directionally consistent ORs), though limited by sample size (Supplementary Figures S3 and S4).

### Interaction analyses suggest predominantly prognostic effects

To distinguish treatment-specific biomarkers from general prognostic indicators in randomized datasets with placebo arms, treatment × endotype interaction terms were evaluated when active vs. placebo arms were available. In UNIFI week-8 mucosal healing models, EPITHELIAL_BARRIER was associated with healing in the placebo arm (OR = 3.66, 95% CI 1.51–8.88), while the active-vs-placebo interaction was not significant (interaction OR = 1.24, 95% CI 0.41–3.80, p = 0.70). Interaction estimates were similarly imprecise for MACROPHAGE_INFLAMMATION (interaction OR = 0.94, 0.38–2.32, p = 0.90) and LYMPHOID_COMPLEMENT_STROMAL (interaction OR = 1.64, 0.74–3.66, p = 0.22). In UNIFI clinical remission, interaction ORs were 0.82 (0.29–2.29, p = 0.70), 0.95 (0.37–2.46, p = 0.92), and 1.76 (0.49–6.35, p = 0.39) for NMF_C1, NMF_C2, and NMF_C3, respectively. In PURSUIT/GSE92415, active-vs-placebo interaction ORs were also non-significant (NMF_C1 OR = 0.57, 0.19–1.75, p = 0.33; NMF_C2 OR = 0.74, 0.19–2.86, p = 0.66; NMF_C3 OR = 1.46, 0.35–6.11, p = 0.61). The placebo-arm epithelial association is most consistent with a baseline healing-capacity axis, whereas the non-significant interaction terms do not support drug-class-specific prediction (Supplementary Figure S2).

### Baseline severity correlations and covariate sensitivity

Baseline severity proxies were available only for a subset of cohorts. MACROPHAGE_INFLAMMATION showed moderate positive correlations with Mayo score or PUCAI in cohorts where these measures were reported (Spearman *ρ* = 0.449–0.524), whereas EPITHELIAL_BARRIER showed inverse correlations with total Mayo score and PUCAI in the pediatric cohort (ρ = −0.354 and −0.341, respectively) and weaker inverse correlation with Mayo endoscopic subscore in GSE73661 (ρ = −0.140; Supplementary Table S5). These patterns suggest that the endotypes partly capture baseline disease activity and mucosal injury state. Where severity proxies were available, covariate-adjusted sensitivity models generally preserved directionality; for example, in GSE73661 severity-adjusted estimates remained directionally consistent for MACROPHAGE_INFLAMMATION (OR = 0.345, 95% CI 0.127–0.937), LYMPHOID_COMPLEMENT_STROMAL (OR = 0.209, 0.069–0.637), and EPITHELIAL_BARRIER (OR = 23.715, 1.807–311.244), while in GSE92415 severity-adjusted effects were directionally consistent but less precise (Supplementary_Tables_All.xlsx, sheet Additional_file_8). Residual confounding remains possible because complete baseline clinical covariates were not available across all cohorts.

### Epithelial and myeloid axes anchor the endotypes

Bulk program scoring linked EPITHELIAL_BARRIER/NMF_C3 to epithelial barrier and goblet programs (Figure 3), while MACROPHAGE_INFLAMMATION/NMF_C1 and LYMPHOID_COMPLEMENT_STROMAL/NMF_C2 tracked inflammatory myeloid and stromal niche proxies in bulk mucosa (Figure 4). These patterns are framed as alignment/localization rather than causality (Figures 3, 4).

**FIGURE 3:**
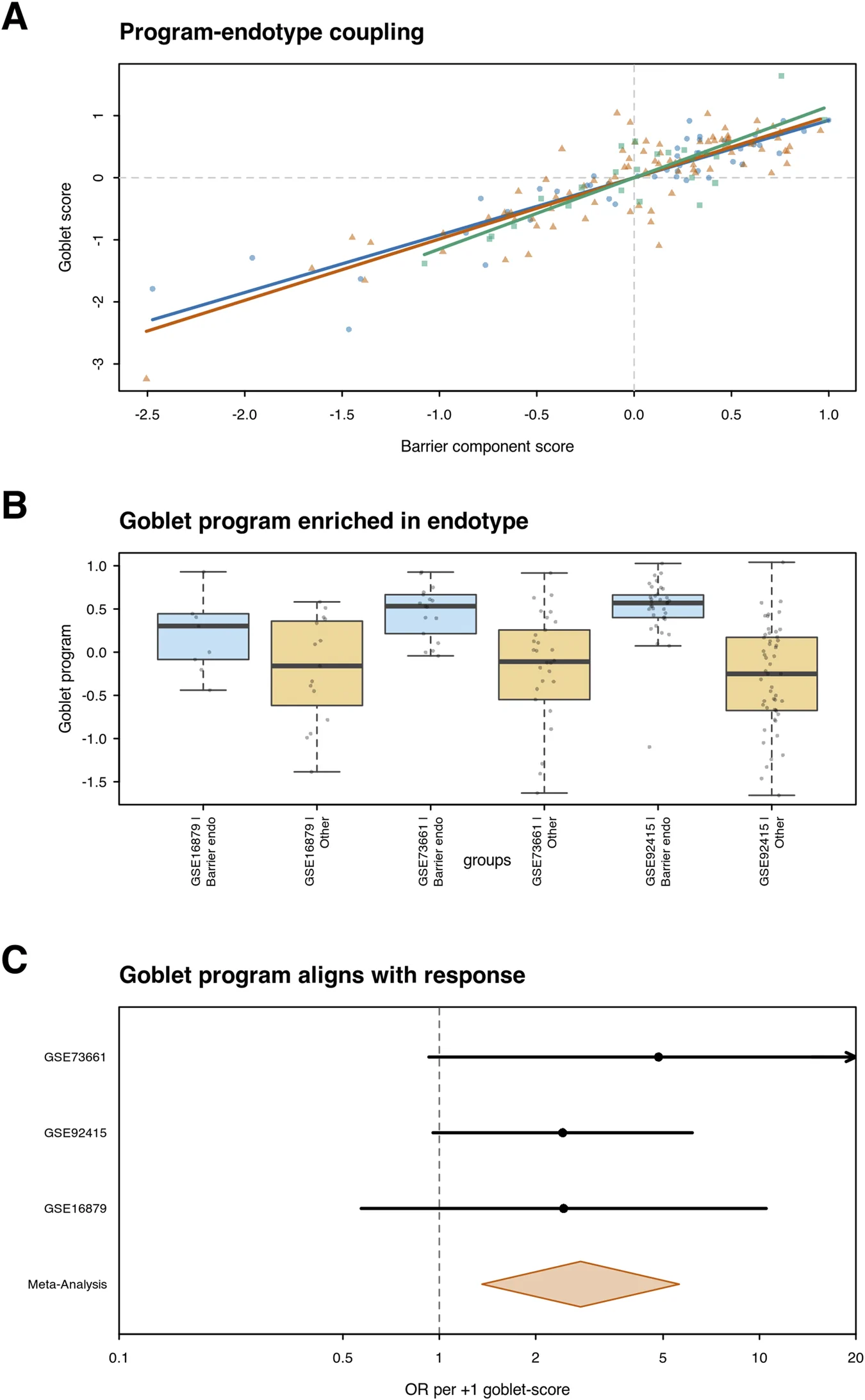
Alignment of response-associated endotypes with epithelial barrier and goblet programs. **(A)** Relationship between the epithelial barrier component score and goblet program score in GSE73661, GSE92415, and GSE16879, with cohort‐specific fitted lines (blue circles, GSE73661; orange triangles, GSE92415; green squares, GSE16879). **(B)** Goblet program scores in samples assigned to the barrier endotype versus other samples within each cohort; points represent individual samples. **(C)** Cohort‐specific odds ratios and 95% confidence intervals for response/healing per one‐unit increase in goblet program score, with the random‐effects pooled summary. The primary endotype‐level synthesis is reported separately in Table 2. The orange diamond represents the pooled estimate and its 95% confidence interval; the right‐pointing arrow indicates a confidence interval extending beyond the plotting limit.

**FIGURE 4:**
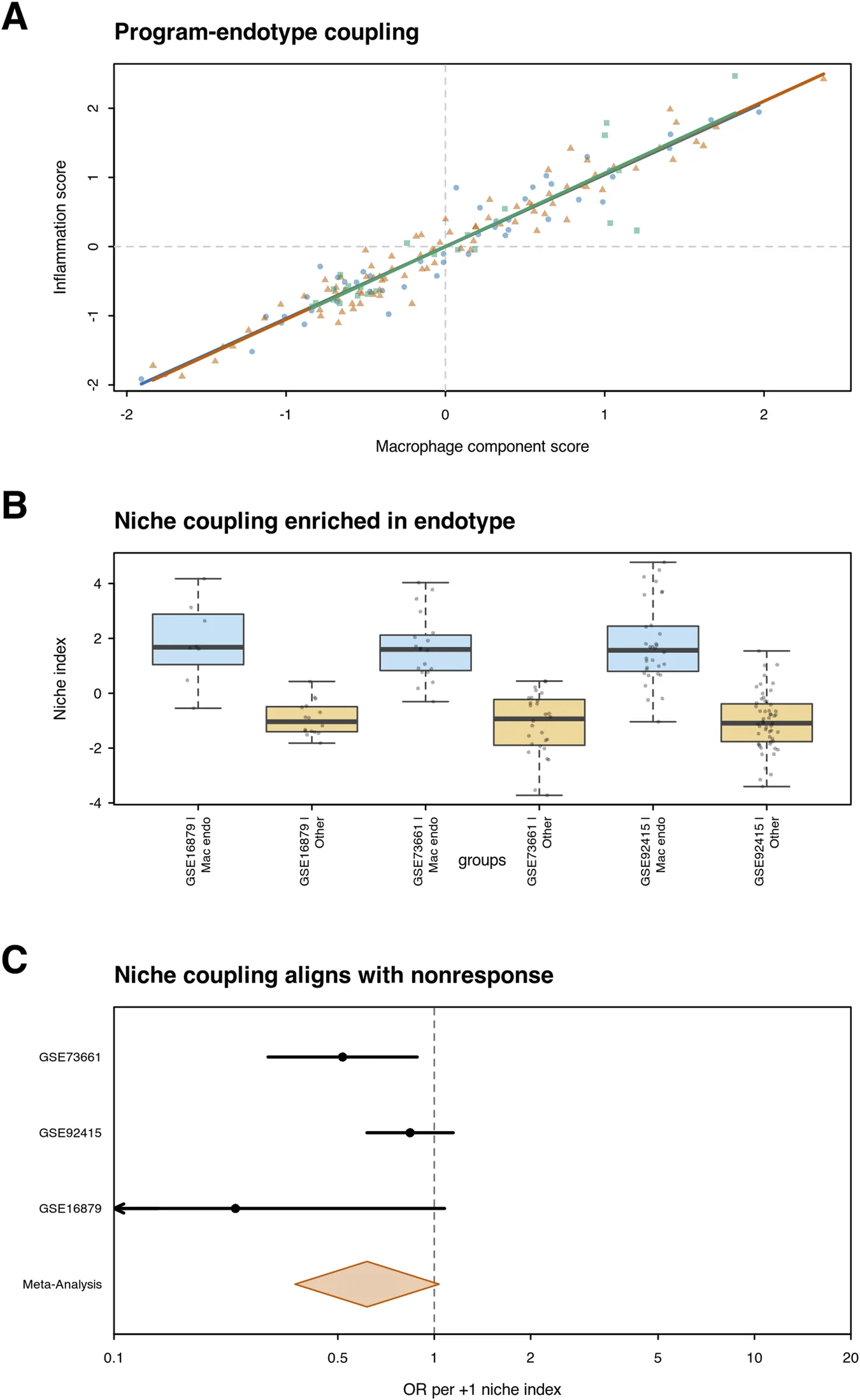
Alignment of the nonresponse endotype with inflammatory myeloid programs and a macrophage niche proxy. **(A)** Relationship between the macrophage component score and inflammatory myeloid program score in GSE73661, GSE92415, and GSE16879, with cohort‐specific fitted lines (blue circles, GSE73661; orange triangles, GSE92415; green squares, GSE16879). **(B)** Niche coupling index in samples assigned to the macrophage endotype versus other samples within each cohort; points represent individual samples. **(C)** Cohort‐specific odds ratios and 95% confidence intervals for response/healing per one‐unit increase in niche coupling index, with the random‐effects pooled summary. Odds ratios below 1 indicate lower odds of response/healing and therefore alignment with nonresponse. The primary endotype‐level synthesis is reported separately in Table 2. The orange diamond represents the pooled estimate and its 95% confidence interval; the left‐pointing arrow indicates a confidence interval extending beyond the plotting limit.

### Single-cell localization to verify cellular provenance (GSE231993)

To ensure that endotypes derived from mixed bulk biopsies reflect genuine cellular programs rather than compositional artifacts, we used an independent UC scRNA cohort (GSE231993) as a high-resolution localization reference. Barrier and goblet program scores were enriched in epithelial compartments, whereas inflammatory myeloid program scores were enriched in myeloid compartments. Mean differences are reported in scaled/log-normalized program-score units. All three localization contrasts were significant by paired sign-flip exact testing (p = 4.88 × 10^−4^; FDR = 0.0026; n = 12 paired samples), supporting the cellular provenance of the bulk signatures (Table 3).

**TABLE 3:** Mechanistic localization corroboration and key evaluation statistics.

Program/analysis	Evidence type	Effect (with uncertainty where available)	p/FDR	n
Barrier program	scRNA localization: epithelial > myeloid	mean diff = 1.421	p = 4.88 × 10^−4^; FDR = 0.0026	12 paired
Goblet program	scRNA localization: epithelial > myeloid	mean diff = 2.002	p = 4.88 × 10^−4^; FDR = 0.0026	12 paired
Inflammatory myeloid program	scRNA localization: myeloid > epithelial	mean diff = 0.502	p = 4.88 × 10^−4^; FDR = 0.0026	12 paired
Therapeutics prioritization	Enrichment for UC-approved therapies	AUC = 0.741	perm p = 6.0 × 10^−4^	12/2,331

### Network-based prioritization provides exploratory perturbation hypotheses

Therapeutic candidates were prioritized against the nonresponse axis (Figure 5) using (i) network proximity from an endotype-associated disease module to drug targets (BioGRID interactome + DrugCentral targets) and (ii) perturbation signature reversal (CREEDS). Non-randomness was assessed using matched negative controls and enrichment toward UC-approved therapies (Table 3). Because the perturbation libraries include non-clinical chemicals and cytotoxic agents, this analysis is interpreted as pathway-level hypothesis generation rather than a direct clinical repurposing list. A clinically focused filtered sensitivity list excluding obvious cytotoxic oncology agents, experimental chemicals, and unclassified perturbagens is provided in Supplementary_Tables_All.xlsx, sheet Additional_file_21; this list is intended only to improve interpretability of the ranking, not to nominate treatments for clinical use.

**FIGURE 5:**
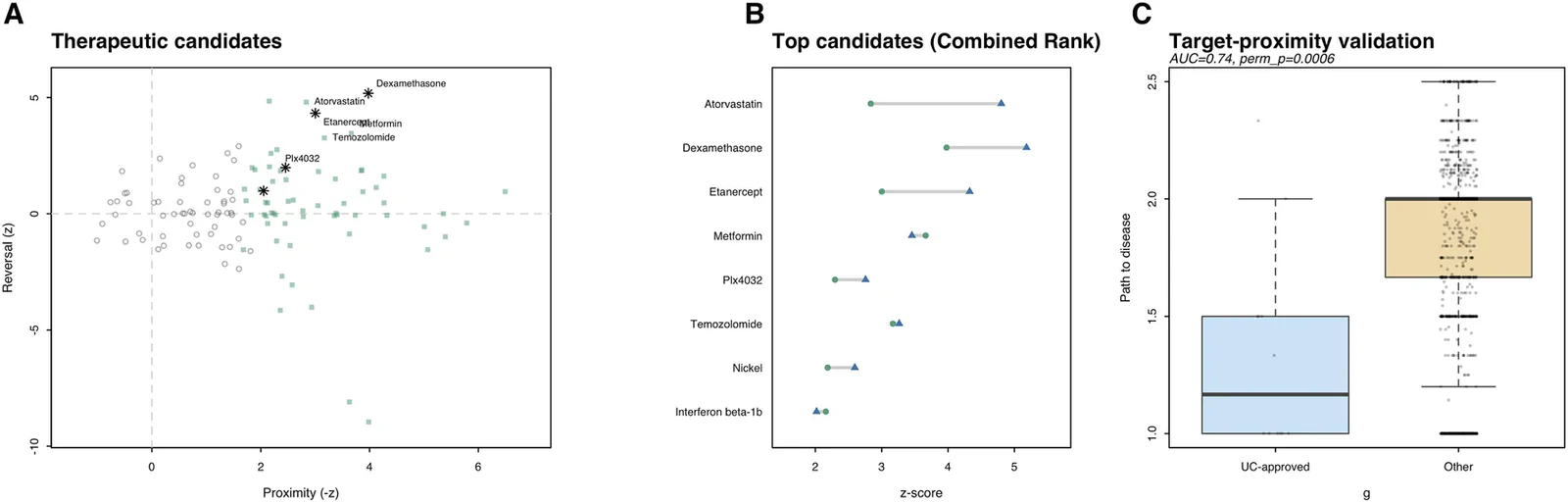
Therapeutic prioritization integrating network proximity and perturbation‐signature reversal. **(A)** Candidate agents plotted by network‐proximity score (–z) and perturbation‐signature reversal score (z). Symbols distinguish agents meeting an FDR threshold of 0.10 for proximity only (green squares), reversal only (blue triangles), both (orange circles), or neither (open gray circles); black stars mark UC control agents. **(B)** Top eight agents ordered by combined rank, with network‐proximity scores shown as green circles and reversal scores as blue triangles. **(C)** Mean shortest‐path distance to the disease module for UC‐approved therapies versus other agents in the target‐based interactome analysis (BioGRID interactome and DrugCentral targets); UC-approved enrichment was assessed by permutation testing (AUC = 0.741; permutation p = 6.0 × 10–4; Table 3). These rankings are hypothesis‐generating.

### Cohort-heldout prediction performance beyond ROC

External prediction evaluation within anti-TNF cohorts used leave-one-cohort-out training/testing (cohort-level holdout) and reported discrimination and calibration. AUC ranged from 0.717 to 0.758, but calibration was inconsistent, with slopes from 0.35 to 0.87 and ECE values of 0.177–0.202. Decision-curve analysis suggested exploratory net benefit over treat-all and treat-none strategies across threshold ranges of 0.05–0.60 for GSE12251, 0.05–0.90 for GSE16879, and 0.20–0.90 for GSE73661 (Supplementary Table S8). Because these ranges were derived from small held-out cohorts and calibration was variable, absolute probability estimates should not be used for individual clinical decision-making without prospective recalibration (Figure 6).

**FIGURE 6:**
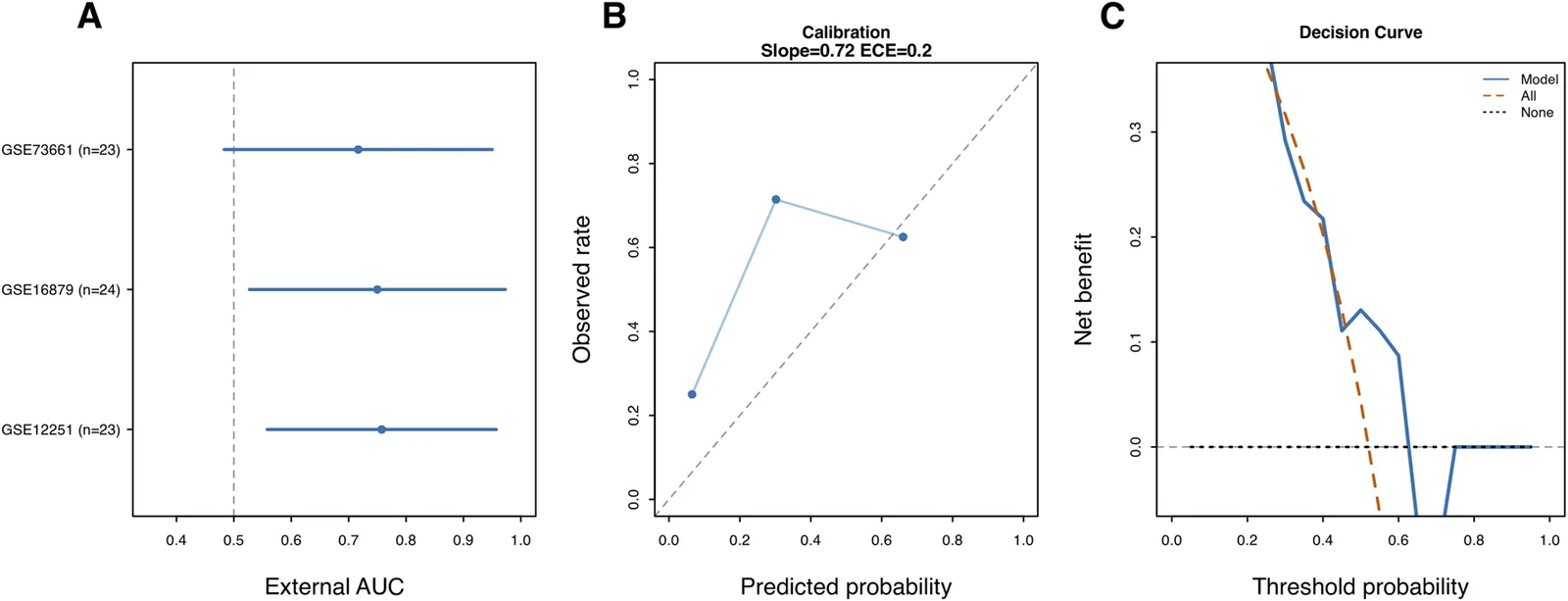
External prediction evaluation beyond ROC (leave‐one‐cohort‐out): discrimination, calibration, and decision curves. **(A)** External AUC estimates with 95% confidence intervals for the held-out GSE12251, GSE16879, and GSE73661 cohorts. **(B)** Calibration in the held-out GSE12251 cohort, with observed response rates plotted against mean predicted probabilities; the dashed diagonal indicates ideal calibration (calibration slope = 0.72; expected calibration error = 0.20). **(C)** Decision‐curve analysis in the same held‐out GSE12251 cohort, comparing the model with treat‐all and treat‐none strategies across threshold probabilities. For panels B and C, training used the pooled GSE73661 and GSE16879 cohorts.

## Discussion

Across public UC cohorts spanning distinct mechanisms of action, baseline mucosal endotypes recapitulated across studies and were associated with response and mucosal healing. Random-effects cross-cohort synthesis supported an EPITHELIAL_BARRIER axis associated with improved outcomes, while MACROPHAGE_INFLAMMATION and LYMPHOID_COMPLEMENT_STROMAL were associated with lower odds of response/healing. The cross-therapy consistency of the epithelial barrier signal is compatible with a common healing-substrate model: regardless of upstream immune blockade, baseline epithelial barrier integrity may represent a permissive state for achieving mucosal healing, whereas baseline inflammatory myeloid and lymphoid/stromal remodeling may reflect a refractory tissue environment that is harder to remodel within typical trial windows. These interpretations remain observational. Epithelial barrier integrity could also be a consequence or correlate of less severe baseline disease rather than an independent driver of treatment response, and the baseline cross-sectional design cannot establish causality.

In datasets with placebo arms, treatment × endotype interaction testing did not provide consistent evidence of treatment specificity, supporting interpretation as outcome-associated stratifiers rather than strictly drug-class-specific predictive biomarkers. Clinically, such biologic-agnostic axes could be evaluated at diagnostic/assessment endoscopy; however, clinical utility will require prospective validation with standardized endpoints, harmonized sample handling, and model recalibration before any individual-level risk estimate is used in practice.

The endotype axes mapped to epithelial barrier/goblet programs and inflammatory myeloid programs. Localization was supported by bulk program scoring and corroborated in an independent scRNA cohort, where barrier and goblet programs localized to epithelial cells and inflammatory myeloid programs localized to myeloid cells. This compartmental pattern is consistent with recent single-cell and spatial studies of UC mucosal remodeling. (Pavlidis et al., 2022; Parikh et al., 2019; Mennillo et al., 2024).

Effect sizes were reported with uncertainty and heterogeneity, and sensitivity checks were used to guard against cohort-specific artifacts. The GSE73661 epithelial estimate remained directionally consistent after bias-reduced logistic regression but was still imprecise; the five-study leave-one-study-out summary remained significant after excluding GSE73661, which supports the pooled signal but does not remove sparse-event uncertainty. External prediction evaluation summarized performance beyond discrimination alone, including calibration and decision-curve analysis (Vickers and Elkin, 2006). The observed AUC values should therefore be interpreted as evidence of signal transferability, not as a deployable clinical prediction model. A prospective study would need to perform logistic recalibration or refit calibration parameters in the target population before reporting absolute probabilities to clinicians. Therapeutic ranking based on network proximity and perturbation reversal provides a data-driven basis for prioritizing biological hypotheses for functional testing, but these rankings should not be read as treatment recommendations.

This retrospective integration of public cohorts is constrained by heterogeneous endpoints and assessment windows (e.g., 6–10 weeks in biologic trials, week-10 clinical remission in EUCALYPTUS, and week-4 remission in a pediatric cohort), which introduces outcome noise and can dilute component-level associations. Not all UC endpoints have equivalent reliability or clinical meaning; endoscopic healing, histologic healing, clinical response, and symptom-based remission can differ across trials and centers. The endpoint-restricted sensitivity analysis partly addresses this concern, but prospective confirmation should use a prespecified endoscopic healing definition and centralized endpoint adjudication. Platform heterogeneity (microarray and RNA-seq), biopsy site differences, and preprocessing choices add technical variance; within-cohort standardization and random-effects models mitigate but do not eliminate these effects. Baseline sampling sites also differed between cohorts (e.g., sigmoid biopsies in UNIFI versus other colonic segments in GEO studies), and anatomic and microenvironmental gradients along the colon could shift epithelial and myeloid proportions in ways that influence component scoring.

Baseline clinical metadata were inconsistently available, limiting covariate adjustment to a subset of cohorts. Severity correlations indicate that endotypes partly track baseline disease activity, and residual confounding remains possible even where severity-adjusted models were directionally consistent. Dose information was limited to treatment or dose-arm labels in selected datasets; dose escalation, therapeutic drug monitoring, anti-drug antibody status, treatment adherence, concomitant corticosteroid exposure, and post-treatment pharmacodynamic data were generally unavailable. These missing treatment-exposure variables could confound response associations and limit the ability to distinguish true pharmacologic prediction from baseline healing propensity. Several estimates came from small cohorts and remain vulnerable to sparse-data bias despite bias-reduced sensitivity checks. Treatment-interaction analyses did not establish drug-class-specific prediction, and model calibration was insufficient for absolute risk prediction. The perturbation component of the therapeutic-prioritization analysis used broad public signatures, so some signal may reflect reversal of general mucosal injury or stress programs rather than UC-specific drug action. Mechanistic localization remains observational. Prospective cohorts with standardized endpoints, baseline severity capture, dose/exposure data, drug-level and anti-drug-antibody measurements, and orthogonal pharmacodynamic readouts will be needed to test stratification utility and refine refractory-state definitions (Goll et al., 2022; Kulecka et al., 2025; Hyams et al., 2022).

Future validation should proceed in a prospective cohort or biomarker-embedded trial that obtains baseline mucosal biopsies before therapy initiation, computes predefined epithelial-barrier and inflammatory/stromal scores using a locked scoring pipeline, and evaluates both general response stratification and therapy-specific interaction terms. Such a study should prespecify whether the biomarker is intended to estimate overall healing probability or to guide selection among biologic mechanisms. Only after calibration, decision-curve, and treatment-interaction performance are confirmed in an external prospective setting should these endotypes be considered for clinical decision support.

## Conclusion

Baseline mucosal transcriptomic endotypes derived from public cohorts were associated with UC response and mucosal healing across biologic mechanisms and aligned with epithelial barrier/goblet and inflammatory myeloid programs. The findings support biologic-agnostic stratification of baseline healing capacity for prospective testing, but do not establish a drug-specific predictive biomarker or a clinically calibrated risk model.

## Data Availability

All datasets analyzed are publicly available through NCBI GEO and are referenced in the manuscript. The processed output tables required to reproduce the figures, together with analysis code, are available in the Zenodo repository: https://doi.org/10.5281/zenodo.23299546.
