# Supplementary material for: Baseline mucosal transcriptomic endotypes stratify healing capacity and biologic therapy outcomes in ulcerative colitis: an integrative multi-cohort study
Source: Front Pharmacol. 2026 Oct 23;17:1931118. doi: 10.3389/fphar.2026.1931118 (PMC13739513; doi:10.3389/fphar.2026.1931118)
Supplement: Supplementary file 1 [file DataSheet1.pdf]

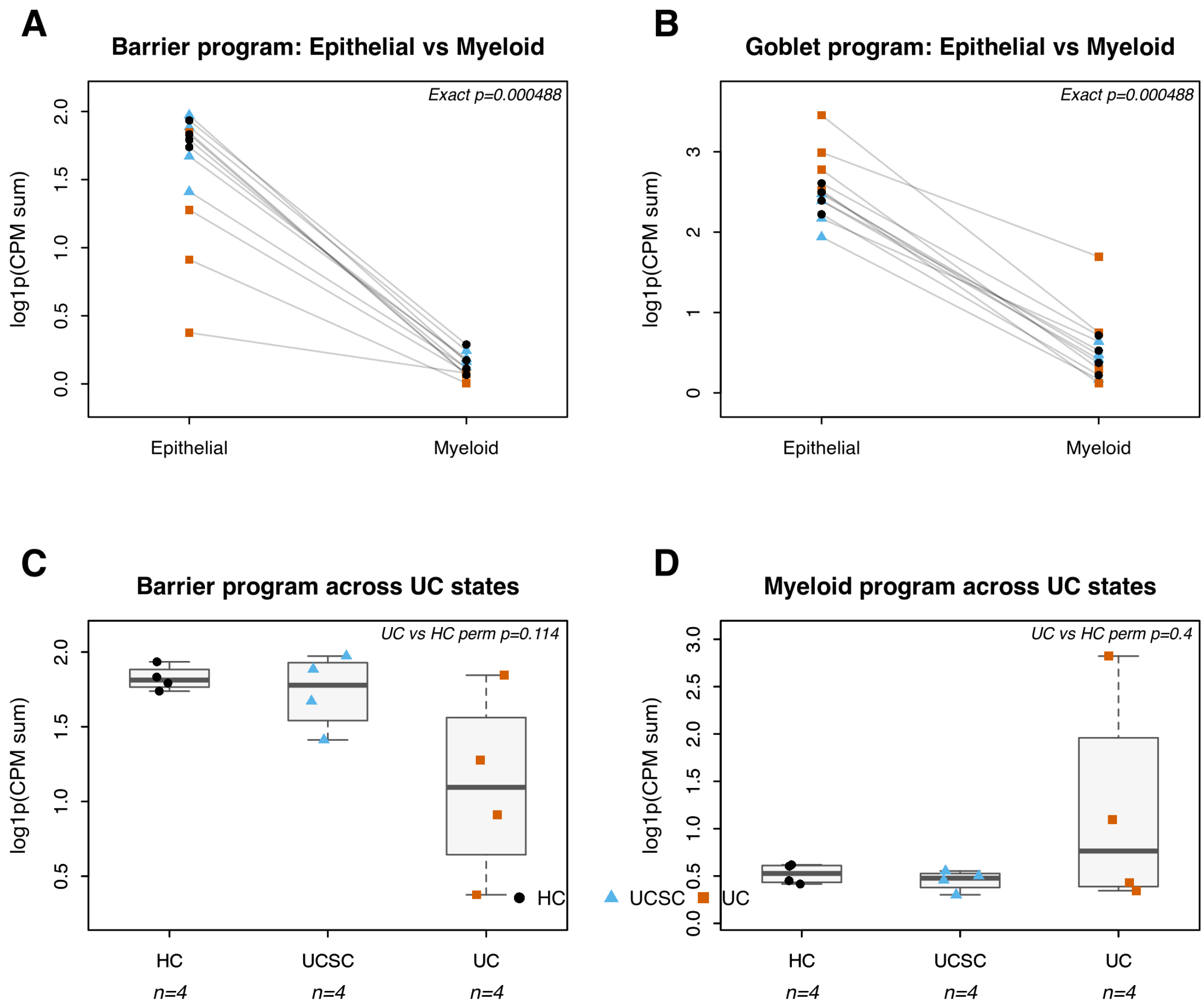

Figure S1. scRNA corroboration of axis localization.

Legend. Independent scRNA corroboration (GSE231993) of endotype axis localization. Barrier and goblet program scores localize to epithelial compartments, whereas inflammatory myeloid program scores localize to myeloid compartments, assessed with paired within-sample contrasts and FDR correction.

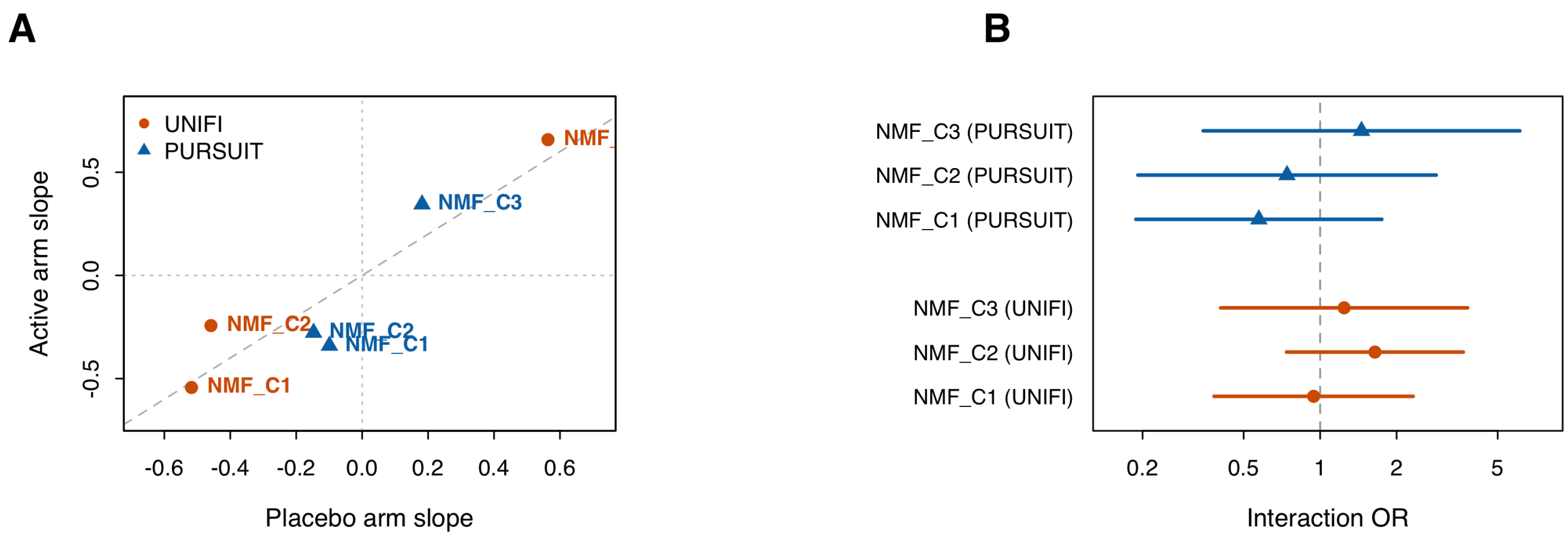

Figure S2. Endotype x treatment interaction tests.

Legend. Endotype x treatment interaction tests in randomized datasets (active vs placebo). Interaction effects are reported as interaction odds ratios (active vs placebo per 1-SD increase in within-cohort standardized endotype score) with 95% confidence intervals.

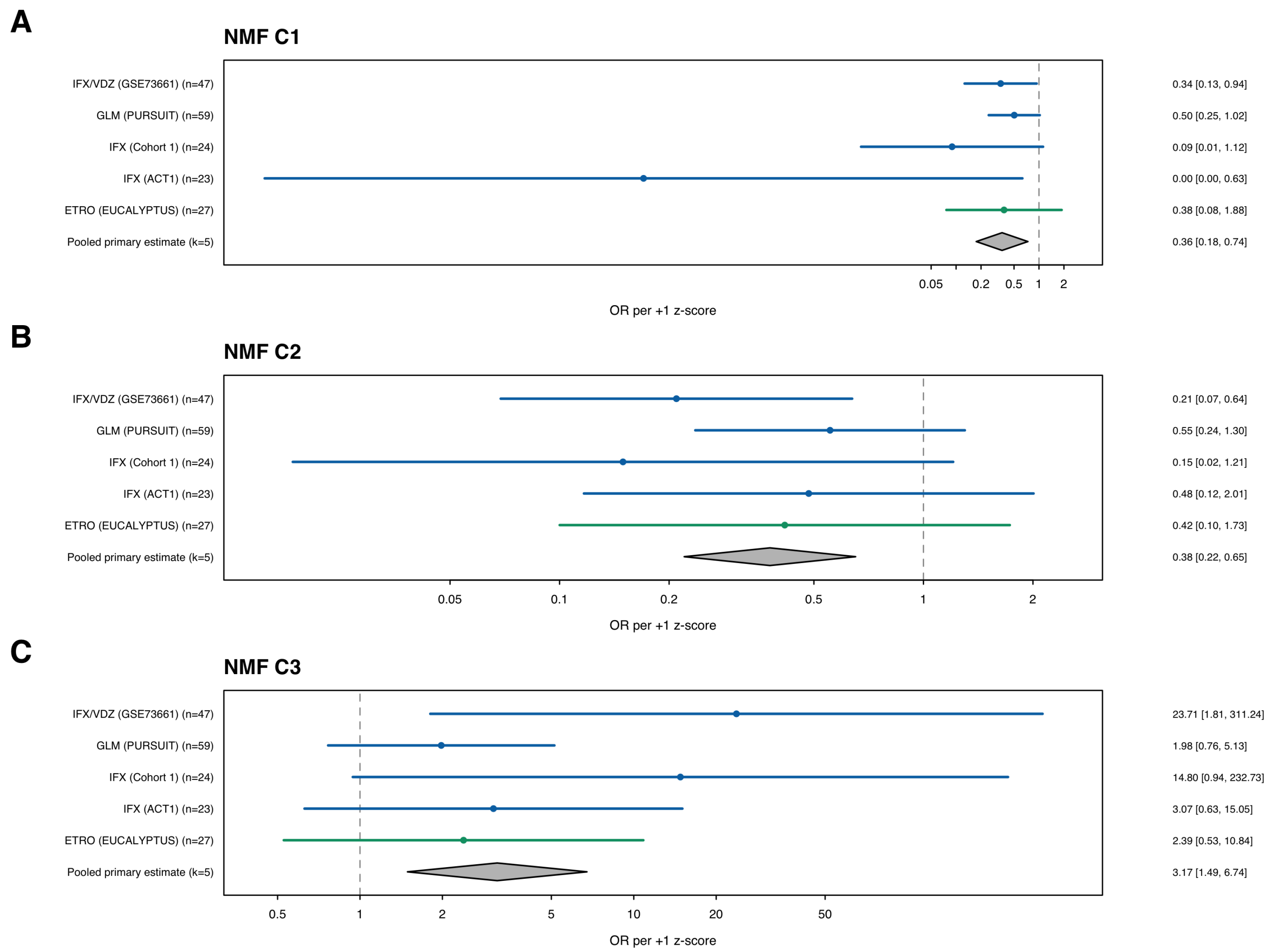

Figure S3. Primary five-study endotype-outcome associations.

Legend. Summary of within-cohort effects and the pooled primary estimate across GSE73661, GSE92415, GSE16879, GSE12251, and GSE72819. UNIFI is not a constituent of this primary pooled estimate and is shown separately in Figure S4.

Numerical display note (A): the GSE12251 estimate is OR = 1.69×10^{-5} (95% CI, 4.56×10^{-10} to 0.63). The original two-decimal labels round these very small positive values to 0.00; the values are not zero. Statistical estimates are unchanged.

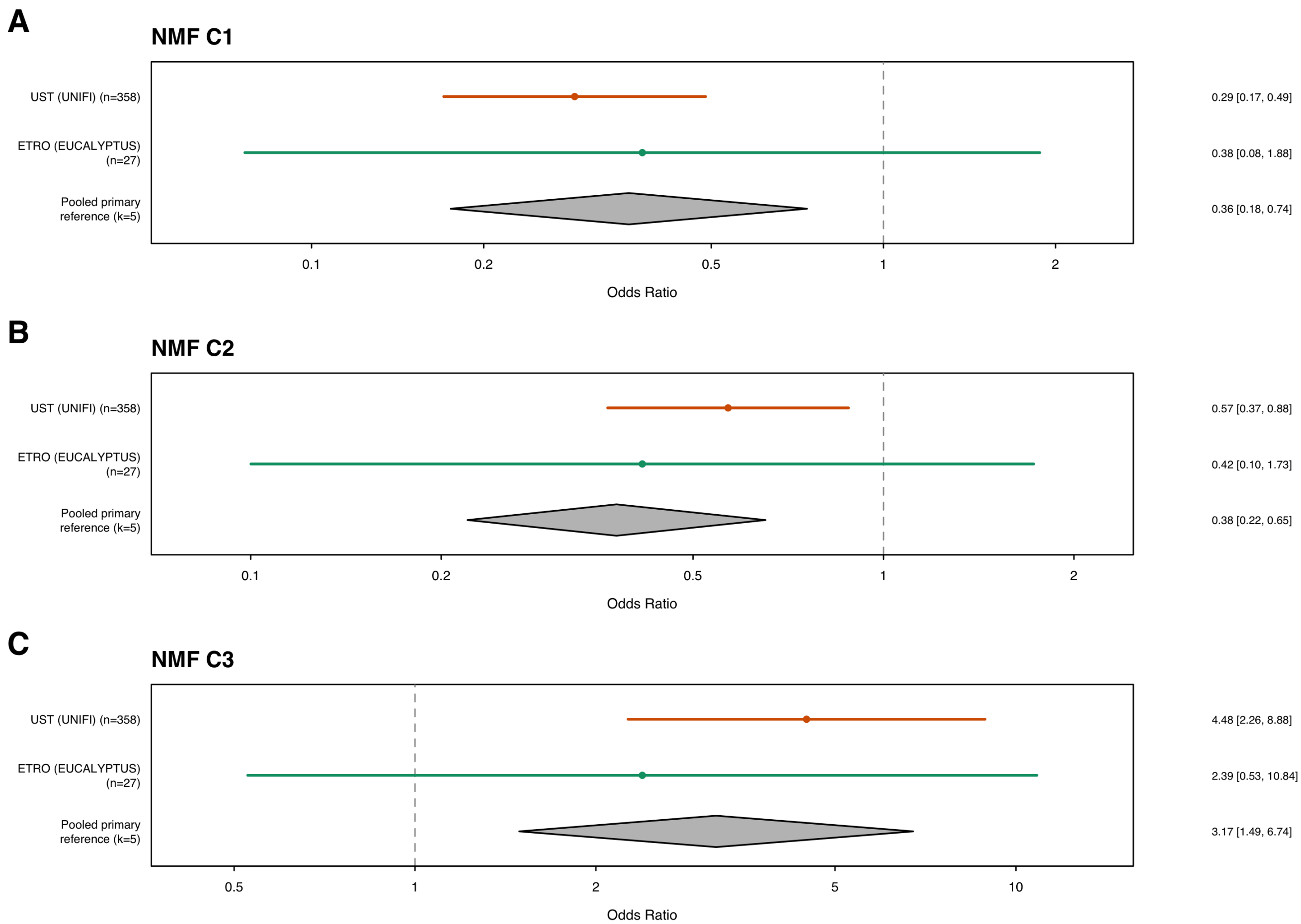

Figure S4. Focused cross-mechanism evaluation.

Legend. Cohort-specific odds ratios with 95% confidence intervals are shown alongside the primary integrated reference, highlighting direction and uncertainty in cross-mechanism cohorts (UNIFI UST and EUCALYPTUS ETRO).

A

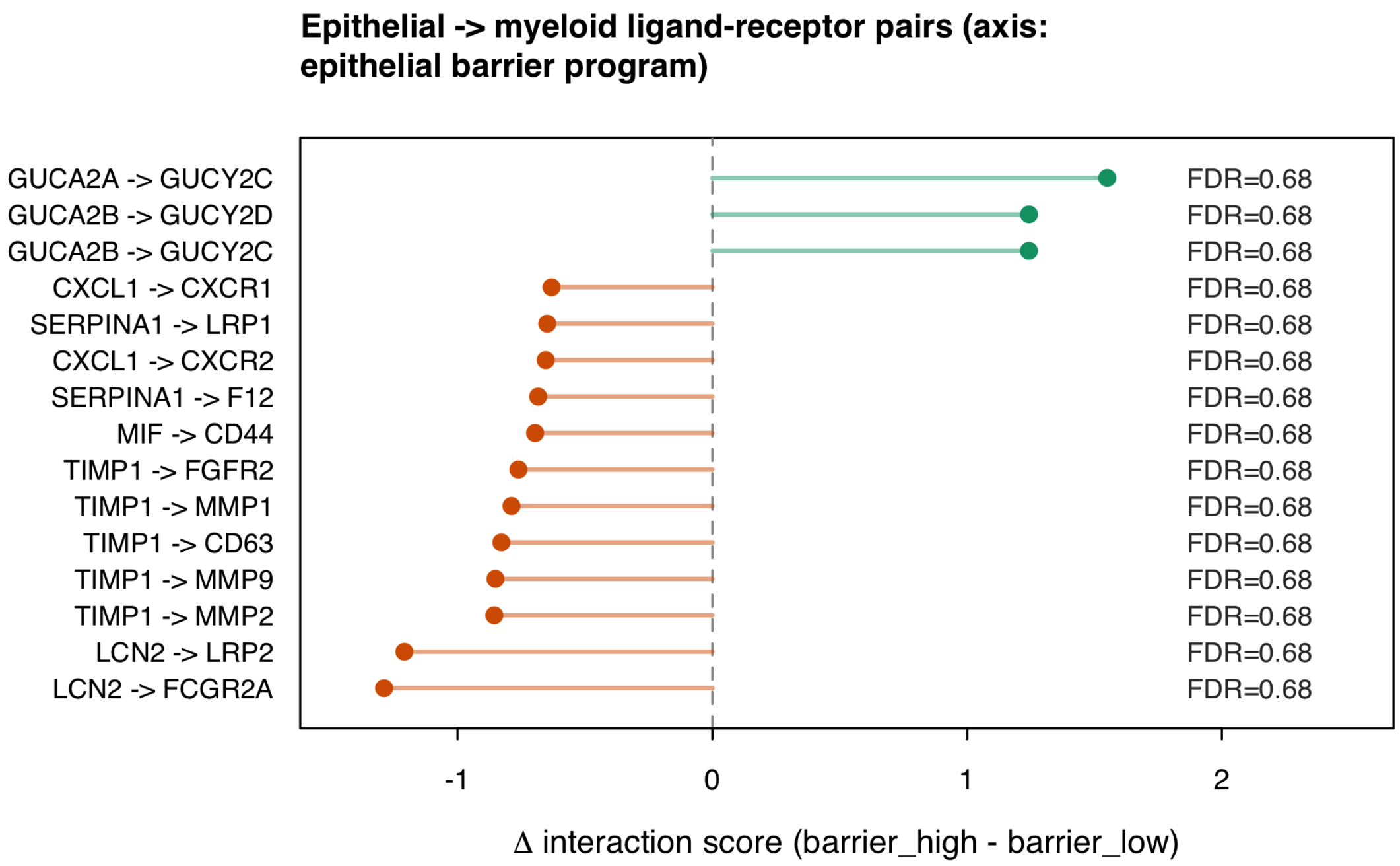

B

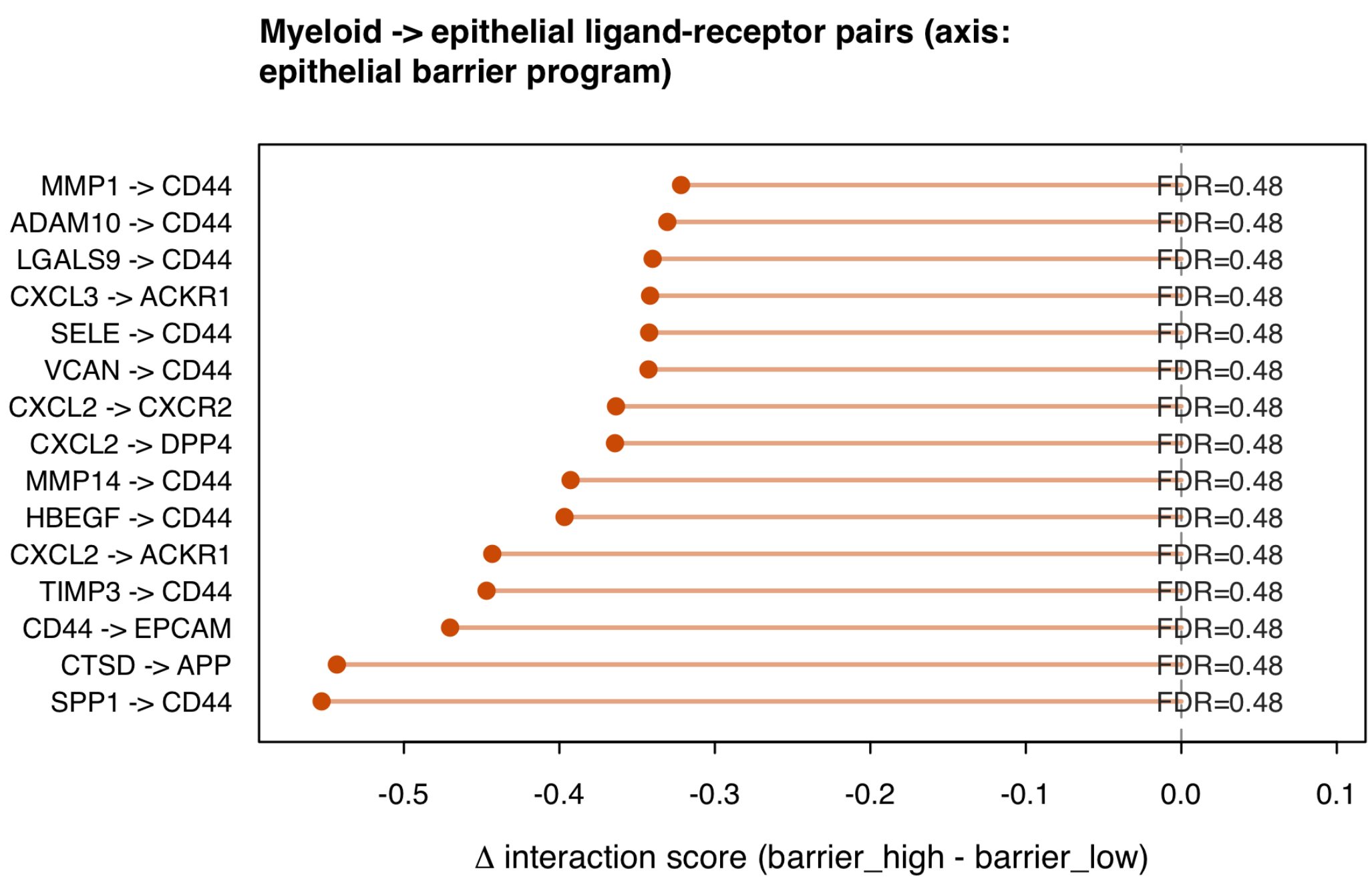

Figure S5. scRNA epithelial-myeloid ligand-receptor communication along the epithelial barrier axis (GSE231993). Legend. For each ligand-receptor pair, interaction scores are computed at the sample level (epithelial to myeloid and myeloid to epithelial directions) and summarized as mean delta interaction score (barrier_high minus barrier_low). No tested ligand-receptor pair reached FDR significance in either direction; this analysis is therefore presented as descriptive context rather than inferential support.
